# Efficacy and safety of remimazolam for non-obese patients during anesthetic induction in cardiac surgery: study protocol for a multicenter randomized trial

**DOI:** 10.1186/s13063-022-06965-8

**Published:** 2022-12-07

**Authors:** Hong Yu, Hong-Mei Liu, Ping Li, Hai Yu, Bin Liu, Peng Liang

**Affiliations:** 1 Department of Anesthesiology, West China Hospital, Sichuan University, No.37 Guoxue Alley, Chengdu, 610041 Sichuan China; 2 Department of Anesthesiology, Wu’an First People’s Hospital, Wu’an, 056300 China; 3 Day Surgery Center, West China Hospital, Sichuan University, No.37 Guoxue Alley, Chengdu, 610041 Sichuan China

**Keywords:** Randomized controlled trial, Remimazolam tosilate, Midazolam, Etomidate, Cardiac surgery, Anesthetic induction

## Abstract

**Background:**

Valvular heart disease remains common in both developed and developing countries, and it requires timely surgical treatment when necessary. However, the stability of hemodynamics during anesthesia induction in patients undergoing valve replacement surgery is difficult to maintain due to their impaired cardiac function. Remimazolam, a novel and ultrashort-acting intravenous sedative-hypnotic, may be beneficial to stable hemodynamics, but the evidence is limited. Therefore, this study aims to evaluate the effect of remimazolam induction on hemodynamics compared with midazolam and etomidate in patients undergoing valve replacement surgery.

**Methods:**

This is a prospective, multicenter randomized controlled trial (RCT). Three hundred and sixty-three non-obese adult patients aged 45 to 80 years old undergoing valve surgery with cardiopulmonary bypass will be randomly allocated to receive remimazolam tosilate, midazolam, or etomidate during anesthetic induction. The primary outcome is the incidence of hypotension within 20 min after the administration of investigated drugs. The hypotension is defined as systolic blood pressure (SBP) < 90 mmHg or a 30% reduction in SBP from baseline or the application of vasoactive drugs. Secondary outcomes include incidence of successful sedation, time to successful sedation, incidence of delirium and postoperative low cardiac output syndrome within 7 days after surgery, hospital mortality, mechanical ventilation time, ICU length of stay, and hospital length of stay.

**Discussion:**

To our knowledge, this is the first prospective RCT to investigate the efficacy and safety of remimazolam induction in adult cardiac surgery compared with midazolam and etomidate. This study will provide important information on the application of remimazolam in cardiac surgery in the future.

**Trial registration:**

Chinese Clinical Trial Registry chictr.org.cn ChiCTR2100050122. Registered on August 16, 2021.

**Supplementary Information:**

The online version contains supplementary material available at 10.1186/s13063-022-06965-8.

## Administrative information

**Table:** 

Title {1}	Efficacy and safety of remimazolam for non-obese patients during anesthetic induction in cardiac surgery: study protocol for a multicenter randomized trial
Trial registration {2a and 2b}	Chinese Clinical Trial Registry ID: ChiCTR2100050122, August 16, 2021
Protocol version {3}	October 2020, version 1.1
F unding {4}	None
Author details {5a}	Hong Yu, MD Department of Anesthesiology, West China Hospital, Sichuan University, Chengdu 610041, China. happyjia1990@foxmail.com Hong-Mei Liu, MSc Department of Anesthesiology, West China Hospital, Sichuan University, Chengdu 610041, China. 18102429203@163.com Ping Li, MSc Department of Anesthesiology, Wu’an First People’s Hospital, Wu’an 056300, China. 595950583@qq.com Hai Yu, MD Department of Anesthesiology, West China Hospital, Sichuan University, Chengdu 610041, China. yuhai@scu.edu.cn Bin Liu, MD Department of Anesthesiology, West China Hospital, Sichuan University, Chengdu 610041, China. liubinhxyy@163.com. Corresponding author Peng Liang, MD Department of Anesthesiology, West China Hospital, Sichuan University, Chengdu 610041. Day Surgery Center, West China Hospital, Sichuan University, Chengdu 610041, China. liangpeng_world@foxmail.com. Corresponding author
Name and contact information for the trial sponsor {5b}	The trial sponsor is the West China Hospital of Sichuan University, Chengdu, China. Postal address: No.37 Guoxue Alley, Chengdu 610041, Sichuan, China. Tel: + 862885423592, Fax: 86–28-85423593
Role of sponsor {5c}	The trial sponsor has a regulatory role and will play a part in study design; collection, management, analysis, and interpretation of data; writing of the report; and the decision to submit the report for publication

## Introduction

### Background and rationale {6a}

Valvular heart disease (VHD) remains common in both developed and developing countries [1, 2], and it keeps a major contributor to physical dysfunction and decreased quality of life [3]. Timely surgical management can mitigate the deterioration to heart failure, disability, and death [4]. However, the stability of hemodynamics of patients with VHD is difficult to maintain due to their varieties of underlying cardiac dysfunction, especially during anesthesia induction when adrenergic tone acutely declines [5, 6].

Remimazolam is a novel and ultrashort-acting intravenous sedative-hypnotic, which mainly acts on the GABA-A receptor [7]. Unlike midazolam, remimazolam has the advantages of stable hemodynamics and mild respiratory inhibition [7–11], making it a potential drug for induction in cardiac surgery anesthesia [12–15]. Midazolam is one of the most frequently used sedatives in cardiac anesthesia. However, midazolam has been reported to be associated with a high incidence of hypotension in patients with cardiac disease and undergoing colonoscopy [16, 17]. Etomidate is another alternative sedative for patients with cardiovascular diseases due to its remarkably stable cardiorespiratory profile [18, 19]. However, etomidate can result in lower cortisol levels and higher adrenal insufficiency incidence compared with midazolam or propofol [18, 20]. So far, there is no evidence for the most appropriate sedative for cardiac surgery anesthesia.

### Objectives {7}

Therefore, our study aims to evaluate the effect of remimazolam induction on hemodynamics compared with midazolam and etomidate in patients undergoing valve replacement surgery. We hypothesized the superiority of remimazolam in the incidence of hypotension compared with midazolam and its non-inferiority compared with etomidate during the induction of cardiac anesthesia.

### Trial design {8}

This study is a prospective, parallel group, three-arm, multicenter randomized controlled trial (RCT). Patients will be allocated in a 1:1:1 ratio to one of the three groups: the remimazolam group (R group), the midazolam group (M group), or the etomidate group (E group).

## Methods

### Study setting {9}

This study will take place in four tertiary hospitals in China including West China Hospital of Sichuan University, Henan Provincial Chest Hospital, Panzhihua Central Hospital, and the Third People’s Hospital of Chengdu upon obtaining ethical approval and written informed patient consent. This trial protocol was written following the Standard Protocol Items: Recommendations for Interventional Trials (SPIRIT) checklist (Additional file 1).

### Eligibility criteria {10}

#### Inclusion criteria

Male and female non-obese patients aged 45 to 80 years are eligible to enter the study if they are scheduled to undergo selective valve surgery with cardiopulmonary bypass (CPB). In addition, they have an ASA physical status score of III or IV, with New York Heart Association (NYHA) class III–IV and a body mass index (BMI) of 18 to 30 kg/m^2^. ***Exclusion criteria.***

Patients meeting one or more criteria listed below will be excluded from this trial:

1. Coma, hypovolemia, or shock with vasoactive drug administration
2. General anesthesia combining with other anesthesia methods (such as epidural anesthesia or spinal anesthesia)
3. Acute heart failure, unstable angina pectoris, myocardial infarction in the last 6 months
4. Infectious heart diseases (such as myocarditis or endocarditis) or sepsis
5. Severe arrhythmia, such as HR ≤ 50 bpm at rest, third-degree atrioventricular block, frequent ventricular premature beat, QTc: male ≥ 450 ms, female ≥ 470 ms
6. Uncontrolled hypertension with antihypertensive drugs [systolic blood pressure (SBP) ≥ 160 mmHg when sitting and/or diastolic blood pressure (DBP) ≥ 100 mmHg when sitting]
7. SBP ≤ 90 mmHg when sitting
8. Abnormal coagulation function (PT, INR, or APTT ≥ 1.5 × upper limit of normal [ULN])
9. Anemia (hemoglobin ≤ 90 g/L) or decreased platelet (platelet ≤ 80 × 10.^9^/L)
10. Abnormal hepatic function (aspartate aminotransferase [AST] or alanine aminotransferase [ALT] ≥ 2.5 × ULN, or total bilirubin ≥ 1.5 × ULN)
11. Abnormal renal function (blood urea nitrogen ≥ 1.5 × ULN or serum creatine exceeds ULN)
12. Hyperglycemia (blood glucose ≥ 11.1 mmol/L)
13. History of alcoholism or drug abuse in the last 2 years
14. Existing mental illness, taking psychotropic drugs for a long time, or cognitive dysfunction
15. Pregnant or lactating women
16. Known contraindication to benzodiazepines, opioids, propofol, muscle relaxants, or investigated drugs
17. Patients with anticipated difficult airway
18. Participation in other trials in the last 3 months
19. Patients with myasthenia gravis
20. Other circumstances deemed by the researchers as inappropriate to participate in this study

### Who will take informed consent? {26a}

One of the trial members who have been trained for the study procedure will screen potential participants according to the inclusion and exclusion criteria, explain the purpose of the study thoroughly, and obtain informed consent (Additional file 2) on the day before the surgery.

### Additional consent provisions for collection and use of participant data and biological specimens {26b}

There is no plan for additional analyses using participant data or biological specimen.

### Interventions

#### Explanation for the choice of comparators {6b}

Midazolam and etomidate are commonly used during cardiac anesthesia induction. And remimazolam is a novel intravenous sedative-hypnotic, and its effect on hemodynamics during cardiac surgery remains uncertain. Thus, we compare the effect of remimazolam with another two drugs on the incidence of hypotension during anesthesia induction.

### Intervention description {11a}

#### Study drug administration

During anesthesia induction, patients in the R group will receive 0.2 mg/kg remimazolam tosilate within 3 min. If the BIS value cannot be ≤ 60 in 3 min, then another 0.1 mg/kg remimazolam tosilate will be added in the following 2 min. Patients in the M group will receive 0.15 mg/kg midazolam within 3 min, and another 0.05 mg/kg midazolam will be employed if the BIS value cannot be ≤ 60 in 3 min. Patients in the E group will receive 0.2 mg/kg etomidate within 3 min, and another 0.05 mg/kg etomidate will be administrated if the BIS value cannot achieve ≤ 60 in 3 min. At the same time, a total of 0.6–1.0 μg/kg sufentanil will be administrated in three groups. When the BIS value < 70 or patients are unresponsive to mild prodding or shaking, cis-atracurium (0.2–0.3 mg/kg) or rocuronium (0.6–1 mg/kg) will be added. If the BIS value cannot achieve ≤ 60 in 5 min after the beginning of the study drug administration, rescue sedative medications with no restriction will be administrated. Endotracheal intubation will be accomplished when BIS maintains 45–55 and the TOF value equals to zero.

#### Anesthesia and CPB management

All patients will be monitored continuously with bispectral index (BIS) electrode, electrocardiogram, pulse oximetry, invasive arterial pressure with pulse index continuous cardiac output (PiCCO) system, central venous pressure (CVP), body temperature, end-tidal partial pressure of carbon dioxide, and train-of-four (TOF) simulation. Anesthesia induction will be conducted according to the group assignment. Anesthesia will be maintained by utilizing propofol or volatile anesthetics (sevoflurane or desflurane) with remifentanil (0.1–0.2 μg/kg/min) to keep a BIS value between 40 and 60. All patients will receive standard CPB management and will be received standard ICU care after surgery.

### Criteria for discontinuing or modifying allocated interventions {11b}

In the case where there is a withdrawal of consent to continue in this study or the patient is allergic to midazolam, etomidate, remimazolam, or other anesthetics during surgery, the patients will be dropped from this study.

### Strategies to improve adherence to interventions {11c}

Not applicable. Interventions in this study will be completed during anesthesia and without the cooperation of patients.

### Relevant concomitant care permitted or prohibited during the trial {11d}

There are no specific restrictions of concomitant care during this study.

### Provisions for post-trial care {30}

Not applicable. Patients will be managed according to routine practice without other post-trial care.

### Outcomes {12}

#### Primary outcome

The primary outcome is the incidence of hypotension within 20 min after the investigated drug administration. The hypotension is defined as SBP < 90 mmHg or a 30% reduction in SBP from baseline or the application of vasoactive drugs.

#### Secondary outcomes

Secondary outcomes include the following: (1) incidence of successful sedation, defined as BIS value ≤ 60 within 5 min after the investigated drug administration, without needing rescue sedative medications [21]; (2) time to successful sedation, defined as the time from initial drug injection to BIS value ≤ 60; (3) postoperative delirium within 7 days after surgery; (4) hospital mortality, incidence of postoperative LCOS, mechanical ventilation time, ICU LOS, and hospital LOS.

### Participant timeline {13}

**Fig. 1:**
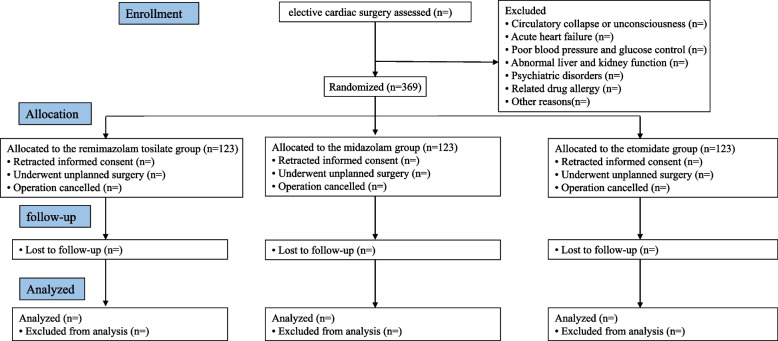
Flow chart

The schedule of trial enrollment, allocation, interventions, and assessments is shown in Table 1 and Fig. 1.

**Table 1:** The SPIRIT figure of this trial

	Study period									
	Enrollment	Allocation	Intervention	Post-intervention						
Time point	Preoperative visit	Allocation	During induction until 20 min after induction	POD1	POD2	POD3	POD4	POD5	POD6	POD7
Enrollment										
Eligibility screen	×									
Informed consent	×									
Demographic date	×									
Allocation		×								
Interventions										
Midazolam			×							
Etomidate			×							
Remimazolam			×							
Surgery and anesthesia date			×							
Assessments			×							
BIS			×							
SBP/DBP/MBP HR/CVP/CO/SVR			×							
RASS			×	×	×	×	×	×	×	×
CAM-ICU			×	×	×	×	×	×	×	×

*POD* postoperative day, *BIS* bispectral index, *SBP* systolic blood pressure, *DBP* diastolic blood pressure, *MBP* mean blood pressure, *HR* heart rate, *CVP* central venous pressure, *CO* cardiac output, *SVR* systematic vascular resistance, *RASS* Richmond Agitation-Sedation Scale, *CAM-ICU* Confusion Assessment Method in the intensive care unit

### Sample size {14}

Based on previous studies, we estimated the incidence of hypotension during induction with remimazolam tosilate, etomidate, and midazolam as 16.7% [12], 13.3% [22], and 44.4% [16], respectively. We hypothesized that remimazolam tosilate was superior to midazolam with superiority marginal = 10% and non-inferior to etomidate with non-inferiority marginal = 10%. A total sample of 336 patients (112 in each group) was required to achieve a power (1–*β*) of 80% and type I *α* error of 0.025. A dropout of 10% was estimated; therefore, the total number of patients was increased to 369 (123 patients per group).

### Recruitment {15}

All patients aged more than 18 with diagnosed VHD, planning for cardiac surgery, will be screened by the investigator in each center daily. Thereafter, verbal and written information regarding the trial will be provided if they meet all inclusion criteria and no exclusion criteria. The recruitment of patients is on-going. Since these four hospitals are high-volume centers, we will be able to identify eligible patients and recruit the required sample size.

### Assignment of interventions: allocation

#### Sequence generation {16a}

The randomization assignment will be generated by a statistician using a block randomization method with a block size of 3 and 6.

#### Concealment mechanism {16b}

Opaque, sealed envelopes generated according to the randomization schedule will be used.

#### Implementation {16c}

On the day of surgery, sequentially numbered opaque envelopes generated according to the randomization schedule will be provided to the attending anesthesiologists.

### Assignment of interventions: blinding

#### Who will be blinded {17a}

Patients, surgical team, anesthesia care providers after induction, care providers in the intensive care unit and ward after surgery, and the investigators assessing outcomes and analyzing data will be blinded to the assignment.

#### Procedure for unblinding if needed {17b}

Not applicable. The attending anesthesiologists who give study drugs during induction will not be blinded to guarantee patients’ safety.

## Data collection and management

### Plans for assessments and collection of outcomes {18a}

All data will be collected in the case report form (CRF) and the following data will be recorded.

#### Baseline characteristics of patients

Demographic data, ASA physical status score, preoperative NYHA grading, diabetes, hypertension, preoperative medication, arrhythmia, chronic obstructive pulmonary disease, dialysis, previous cerebral infarction, hypothyroidism, chronic heart failure, left ventricular ejection fraction (LVEF), cardiac surgery history, EuroScore II score, type of surgery, operation time, extracorporeal circulation time, aortic clamp time, the amount of blood product, and fluid balance will be recorded.

#### Evaluation during anesthesia induction

We will record the BIS value, the time from drug administration to the BIS value ≤ 60, and the dosage of the sedative and sufentanil. Rescue sedative medications and the dosage used will also be recorded if the first sedation fails. The following hemodynamic data will be collected every 30 s for 20 min after the investigated drug administration: SBP, DBP, mean blood pressure, CVP, heart rate, cardiac output (CO), and systemic vascular resistance (SVR). The dosage of vasoactive drugs used within 20 min after induction will also be recorded. Bucking during induction will be recorded and scored as follows: no bucking, mild bucking (1–2 times), moderate bucking (3–4 times), and severe bucking (≥ 5 times).

#### Delirium evaluation and follow-up at discharge

Postoperative cognitive function will be evaluated daily (15:00–17:00) using the Richmond Agitation-Sedation Scale (RASS) score and Confusion Assessment Method in ICU (CAM-ICU) [23] within the first 7 postoperative days. And we also record hospital mortality, postoperative low CO syndrome (LCOS), mechanical ventilation time (from return to ICU after surgery to successful tracheal extubation), ICU length of stay (LOS), and hospital LOS.

### Plans to promote participant retention and complete follow-up {18b}

During the preoperative visit, patients will be fully informed about the intraoperative procedure and postoperative follow-up. And the study investigators who are in charge of follow-up will visit patients daily within the first 7 postoperative days.

### Data management {19}

Data recorded in CRFs will be entered into the Electronic Data Capture (EDC) system by trained nurses from each sub-center study team. Data accuracy will be supervised by the principal investigators (HY, HML, and PL). And all study members will receive protocol and device training (if necessary) before participating in the study to ensure protocol adherence. Data will be stored for at least 3 years after trial termination and publication of the final report.

### Confidentiality {27}

All records of trial participants will be stored using identification numbers and initials in CRF and EDC system and remain confidential to the public.

### Plans for collection, laboratory evaluation, and storage of biological specimens for genetic or molecular analysis in this trial/future use {33}

Not applicable. There exist no biological specimens to be collected in this study.

## Statistical methods

### Statistical methods for primary and secondary outcomes {20a}

Statistical analysis will be performed using SPSS 23.0 software. The continuous data will be described as the mean (SD) or median (interquartile range) and compared using the Student *t* test or the Mann–Whitney *U* test between groups, whereas categorical data will be described as frequencies (percentages) and compared using Pearson *χ*^2^ test or Fisher exact tests. And we will conduct the analysis of variance for repeated measurements with Bonferroni correction to compare the hemodynamic effects between remimazolam and midazolam and between remimazolam and etomidate. To test the superiority of remimazolam tosilate to midazolam and the non-inferiority of remimazolam tosilate to etomidate, one-tailed tests will be performed, and other tests will utilize a two-tailed test. All tests will be conducted with 95% confidence intervals. A *P* value of less than 0.05 will be considered statistically significant.

### Interim analyses {21b}

No interim analyses are planned for this study.

### Methods for additional analyses (e.g., subgroup analyses) {20b}

No subgroup analyses are planned for this study.

### Methods in analysis to handle protocol non-adherence and any statistical methods to handle missing data {20c}

Primary analyses will be performed according to the intention-to-treat principle. Sensitivity analysis will be performed to handle protocol violation and deviation cases using the per-protocol principle. If the continuously collected data (e.g., SBP, DBP, BIS, RASS, CAM-ICU, et al.) is lost at one timepoint during the trial, the most recent data will be analyzed as if it were obtained at that time.

### Plans to give access to the full protocol, participant-level data, and statistical code {31c}

The datasets analyzed during the current study and statistical code are available from the corresponding author on reasonable request, as is the full protocol.

## Oversight and monitoring

### Composition of the coordinating center and trial steering committee {5d}

There are several tertiary hospitals in China participating in this study. The study team has a conference online weekly to discuss the problems in the conduct of the trial. There is no trial steering committee or stakeholder and public involvement group.

### Composition of the data monitoring committee, its role, and reporting structure {21a}

Not applicable. There is no data monitoring committee in this study. Instead, the principal investigator will check the appropriateness of the data and conduction of the trial every 2 weeks.

### Adverse event reporting and harms {22}

According to the drug instruction of remimazolam tosilate, the adverse events related to this drug include hypotension (2.65–6.45%), decreased neutrophil (6.45%), increased serum bilirubin (3.17%), increased serum unconjugated bilirubin, and dizziness (2.12%). But these adverse events will not result in severe consequences. Adverse events related to this study protocol include severe hypotension and failure in sedation. When hypotension occurs, vasoactive drugs will be utilized during anesthesia induction when SBP < 90 mmHg or a 30% reduction from baseline. And when study sedation fails, rescue sedative medications (including propofol, midazolam, or etomidate, etc.) will be used to accomplish anesthesia induction. Adverse events will be treated immediately and reported to the institutional review board. The participants will also be followed up until it is completely resolved or therapy is terminated.

### Frequency and plans for auditing trial conduct {23}

The department of research office and the ethics committee of each center will audit the study annually. And the principal investigator will check the appropriateness of the data and conduction of the trial every 2 weeks.

### Plans for communicating important protocol amendments to relevant parties (e.g., trail participants, ethical committees) {25}

Any protocol amendments will be written into a formal substantial amendment reviewed by the institutional review board of the West China Hospital before application.

### Dissemination plans {31a}

ALL patients will provide written informed consent before the start of any protocol-specified procedures or assessments. The results of the study will be presented at relevant conferences and submitted to international peer-reviewed journals.

## Discussion

The current study will be the first prospective RCT assessing the efficacy and safety of employing remimazolam tosilate for anesthesia induction in patients undergoing cardiac surgery compared with midazolam and etomidate. Our results may show that the infusion of remimazolam tosilate titrated to a BIS value of 60 is superior to midazolam and non-inferior to etomidate on the incidence of hypotension during anesthesia induction.

The primary advantages of remimazolam tosilate include rapid onset of action and minimal depression of cardiorespiratory function, allowing this drug favorable in sedation and general anesthesia. Several clinical cases found that remimazolam can be used to achieve the same appropriate anesthesia management as other existing anesthetics for patients undergoing cardiac surgery [13, 14, 24] or for the patient undergoing non-cardiac surgery with cardiac dysfunction [25]. And two RCTs [12, 15] suggested that remimazolam was safe and effective compared with propofol for induction in cardiac surgery with less hemodynamic fluctuations. However, propofol has a huge potential to cause hypotension, and its incidence could reach more than 70% during general anesthesia induction [26, 27]. It indicates that propofol may be inappropriate for patients with impaired cardiac function, consequently making the benefit of remimazolam on cardiovascular function less convincing when comparing with propofol. Clinical trials comparing remimazolam with sedatives other than propofol during cardiac surgery are necessary and significant. Midazolam is the most frequently used sedative for anesthetic induction with mild adverse events [28], but there was scarce study investigating the hemodynamic change of midazolam in cardiac surgery so far. Besides, etomidate may be a better control drug as it has been proven to be associated with more stable hemodynamics for anesthetic induction in cardiac surgery [18] but with side effects of injection-site pain, myoclonus, and suppression of the adrenocortical axis [20]. Until now, the most optimal sedative for anesthesia induction in cardiac surgery was uncertain. Therefore, we design this study to evaluate the efficacy and safety profile of remimazolam tosilate during cardiac surgery by comparing with midazolam and etomidate. In summary, we anticipate the findings of this study will provide a high-quality evidence on the merit of stabilizing hemodynamics of remimazolam tosilate during anesthetic induction in cardiac surgery.

## Strengths and limitations

This study is, to our knowledge, the first to evaluate the efficacy and safety of utilizing remimazolam tosilate in patients undergoing cardiac surgery. In addition, this is a multicenter trial involving several tertiary hospitals in China, making the results from this study generalizable. This prospective RCT has been carefully designed and will be meticulously implemented in each center. The major limitation of this study is that the definition of hypotension and the characteristics of participants varied in different studies, resulting in the potential difference between the estimated and the actual incidence of hypotension. In addition, we excluded obese patients with BMI > 30 in this study because obese patients often suffer from a combination of hypertension, dyslipidemia, and type 2 diabetes mellitus, also known as the “metabolic syndrome” [29]. Consequently, the pharmacokinetics of obese patients are different from patients with a normal range of BMI. Therefore, to control bias and also to guarantee patients’ safety, we will only recruit patients with BMI between 18 and 30.

## Trial status

The current protocol is version 1.1 and was issued on 1 October 2020. At the time of manuscript submission, the study is in the phase of recruitment. Recruitment has been begun in April 2022 and is expected to be completed by June 2023.

## Supplementary Information

**Additional file 1. **The SPIRIT 2013 checklist of this trial.

**Additional file 2. **The model consent form.

## Data Availability

Any data required to support the protocol can be supplied on request.

## Abbreviations

ALT: Alanine aminotransferase
APTT: Activated partial thromboplastin time
AST: Aspartate aminotransferase
BIS: Bispectral index
BMI: Body mass index
CAM-ICU: Confusion Assessment Method in the intensive care unit
CO: Cardiac output
CPB: Cardiopulmonary bypass
CVP: Central venous pressure
DBP: Diastolic blood pressure
GABA: Gamma-aminobutyric acid
ICU: Intensive care unit
INR: International normalized ratio
LCOS: Low cardiac output syndrome
LOS: Length of stay
LVEF: Left ventricular ejection fraction
NYHA: New York Heart Association
PiCCO: Pulse index continuous cardiac output
PT: Prothrombin time
RASS: Richmond Agitation-Sedation Scale
RCT: Randomized controlled trial
RHD: Rheumatic heart disease
SBP: Systolic blood pressure
SD: Standard deviation
SPIRIT: Standard Protocol Items: Recommendations for Interventional Trials
SVR: Systemic vascular resistance
TOF: Train of four
ULN: Upper limit of normal
