# Supplementary material for: Efficacy and safety of remimazolam for non-obese patients during anesthetic induction in cardiac surgery: study protocol for a multicenter randomized trial
Source: Trials. 2022 Dec 7;23:984. doi: 10.1186/s13063-022-06965-8 (PMC9727858; doi:10.1186/s13063-022-06965-8)
Supplement: Supplementary file 2 — Additional file 2. The model consent form. [file 13063_2022_6965_MOESM2_ESM.docx]

**Efficacy and safety of remimazolam during anesthetic induction in cardiac surgery**

**Informed Consent Form**

Version number: Version 1.0

Research institute: Department of Anesthesiology, West China Hospital of Sichuan University, Chengdu, Sichuan

Director of research： Bin Liu

Contact number： 18980601540

**Version date: December 23^rd^ , 2020**

**Informed Consent Form - Inform**

Dear patients,

We would like to invite you to participate in a multi-center, prospective, randomized, controlled clinical study on the efficacy and safety of remimazolam on the incidence of hypotension during anesthesia induction in cardiac surgery approved by the biomedical ethics committee of West China hospital of Sichuan university. This informed consent will give you a brief introduction of the study. It can help you understand the content of the study, why the study is conducted, and the points of your cooperation. After reading and understanding the following information, please decide whether to participate in the study and whether to sign the informed consent.

**1. Background**

Valvular heart disease remains common in both developed and developing countries and it keeps a major contributor to physical dysfunction, decreased quality of life. Timely surgical management can mitigate the deterioration to heart failure, disability, and death. However, the stability of hemodynamics of patients with VHD is difficult to maintain due to their varieties of underlying cardiac dysfunction, especially during anesthesia induction when adrenergic tone acutely declines.

Remimazolam is a novel and ultrashort-acting intravenous sedative-hypnotic, which mainly acts on GABA-A receptor. Unlike midazolam, remimazolam has the advantages of stable hemodynamicsand mild respiratory inhibition, making it a potential drug for induction in cardiac surgery anesthesia. Etomidate is another alternative sedative for patients with cardiovascular diseases due to its remarkably stable cardiorespiratory profile. However, etomidate can result in lower cortisol levels and higher adrenal insufficiency incidence compared with midazolam or propofol. So far, there is no evidence for the most appropriate sedative for cardiac surgery anesthesia.

The sedatives used in this study and intraoperative monitoring methods involved in the study are common technical methods used in West China hospital, which do not increase your perioperative risk.

**2. Objectives**

To evaluate the effect of remimazolam induction on hemodynamics compared with midazolam and etomidate in patients undergoing valve replacement surgery.

**3. Methods**

We will fully evaluate before participating in the study. If you are eligible for inclusion criterion, you can voluntarily participate in the study and sign this informed consent. And then, you will be randomly assigned to different study groups. Regardless of allocation, there was no increased risk of any accidents or complications. If you do not want to participate in the study, your operation and anesthesia will not be affected.

1. Remimazolam group：Application of remimazolam for sedation during anesthesia induction

2. Midazolam group：Application of midazolam for sedation during anesthesia induction

3. Etomidate group: Application of etomidate for sedation during anesthesia induction

Intraoperative anesthetics, vital signs, and postoperative physical examinations will be recorded.

**4. Study duration**

The interventions in this study will last for 7 day postoperatively.

**5. Risk and benefits**

Sedatives are necessary during anesthesia induction. Remimazolam is a novel and ultrashort-acting intravenous sedative and it has been used in colonoscopy safely. And another two sedatives are commonly applied in clinical practice. Even if you are not involved in this trial, your attending anesthesiologist will also choose one kind of sedatives during the operation. In addition, other anesthesia techniques used in the study are all clinical routine and will not cause additional physical damage.

This study also will not increase the incidence of anesthesia-related risks.

**6.Risk prevention and emergency plan**

If you experience any discomfort during the study, whether related to the study or not, you will be notified to your attending doctors. They will make a judgment and give you appropriate medical treatments.

In the study, careful monitor and evaluate will be taken timely and reasonably to deal with the adverse events, in order to reduce your risk as much as possible. In case of any damage related to the research, we will provide treatment plans, bear the cost of treatment and give you corresponding economic compensation according to relevant regulations of the state.

During the study period, you need to cooperate in the evaluation and follow-up, which will take some of your time and may also cause you trouble or inconvenience.

**7. Sample preservation**

There’s no demand of blood collection. The laboratory examination observed and recorded by the researchers were routine examinations. The samples were stored in accordance with the relevant standards of the clinical laboratory department of the hospital, and were not used for other purposes.

**8. Secrecy**

Your medical records will be kept in the hospital completely, and your medical data will be permanently kept by the medical record department of the hospital. Researchers, ethics committees and health authorities will be allowed to access your medical records. The researchers of this program are not allowed to have access to your medical records. Any public report on the results of this study will not disclose your personal identity. We will, to the extent permitted by law, make every effort to protect the privacy of your personal medical data。

**9. Voluntary principle**

The study is in accordance with " the Criterions for the Quality Control of Clinical Trial of Drugs " and "Helsinki declaration", and has been approved by the medical ethics committee, which will ensure your rights and interests. You have right to refuse to participate, or withdraw at any time during the study, which will not affect your treatment. Considering your best interest, the study may be terminated at any time by the doctor or investigator. You can raise any questions about this study at any time and will receive the corresponding explains. If there is any important new information during the study that may affect your willingness to continue to participate in the study, your doctor will inform you in time.

**10．Acknowledgement**

Thank you for reading the material. If you decide to participate in the study, please inform your doctor who will arrange all the affairs for you. Please keep this information.

**Efficacy and safety of remimazolam during anesthetic induction in cardiac surgery**

**Informed Consent Form - signature**

I have read introduction above and have the right to discuss or raise questions with my doctor about this study. All my questions were answered satisfactorily. I am aware of the risks and benefits. I understand that participation in the study is voluntary and I confirm that I have had sufficient time to consider and understand that:

1. I will abide by the requirements of the instructions, participate in the study voluntarily, and fully cooperate with the researchers to be truthful and honest.

2. The results of the trial are only used for scientific research. Except for relevant national administrative departments, ethics committee of West China hospital, bidding unit, and investigators, my personal data in the study are confidential and will be protected in accordance with the law.

3. I voluntarily participate in this study, understand the possible benefits and risks, comply with the requirements of the program, and fully cooperate with the researchers.

4. My decision in this study is completely voluntary. I can refuse to participate or withdraw from the trial at any time without discrimination or retaliation, and my medical treatment will not be affected.

Patient: Date

I confirm that I have explained the details of this study to the patient, including his rights, possible benefits and risks, and have given him a copy of the signed informed consent.

Researcher: Date

Contact information:

Telephone:

Address:
